# TLR9 Ligand (CpG Oligodeoxynucleotide) Induces CLL B-Cells to Differentiate into CD20^+^ Antibody-Secreting Cells

**DOI:** 10.3389/fimmu.2014.00292

**Published:** 2014-06-16

**Authors:** Hussein Ghamlouch, Hakim Ouled-Haddou, Aude Guyart, Aline Regnier, Stéphanie Trudel, Jean-François Claisse, Vincent Fuentes, Bruno Royer, Jean-Pierre Marolleau, Brigitte Gubler

**Affiliations:** ^1^EA4666, Department of Immunology, Université de Picardie Jules Verne, Amiens, France; ^2^Service d’Hématologie Clinique et Thérapie Cellulaire, Department of Hematology, Centre Hospitalier Régional Universitaire d’Amiens, Amiens, France; ^3^Laboratoire d’Oncobiologie Moléculaire, Department of Molecular Oncobiology, Centre Hospitalier Régional Universitaire d’Amiens, Amiens, France

**Keywords:** chronic lymphocytic leukemia, CpG oligodeoxynucleotide, CD20, antibody-secreting cells, B-cell differentiation, memory B cell, poly/autoreactive IgM

## Abstract

B-cell chronic lymphocytic leukemia (CLL) is the most frequent adult leukemia in the Western world. It is a heterogeneous disease characterized by clonal proliferation and the accumulation of CD5^+^ mature B lymphocytes. However, the normal counterpart from which the latter cells arise has not yet been identified. CD27 expression and gene expression profiling data suggest that CLL cells are related to memory B-cells. *In vitro*, memory B-cells differentiate into plasma cells when stimulated with CpG oligodeoxynucleotide (CpG). The objective of the present study was therefore to investigate the ability of CpG, in the context of CD40 ligation, to induce the differentiation of CLL B-cells into antibody-secreting cells (ASCs). CD20^+^CD38^−^ CLL B-cells were stimulated with a combination of CpG, CD40 ligand and cytokines (CpG/CD40L/c) in a two-step, 7-day culture system. We found that the CpG/CD40L/c culture system prompted CLL B-cells to differentiate into CD19^+^CD20^+^CD27^+^CD38^−^ASCs. These cells secreted large amounts of IgM and had the same shape as plasma cells. However, only IgMs secreted by ASCs that had differentiated from unmutated CLL B-cells were poly/autoreactive. Class-switch recombination (CSR) to IgG and IgA was detected in cells expressing the activation-induced cytidine deaminase gene (*AICDA*). Although these ASCs expressed high levels of the transcription factors *PRDM1* (BLIMP1), *IRF4*, and *XBP1s*, they did not downregulate expression of *PAX5*. Our results suggest that CLL B-cells can differentiate into ASCs, undergo CSR and produce poly/autoreactive antibodies. Furthermore, our findings may be relevant for (i) identifying the normal counterpart of CLL B-cells and (ii) developing novel treatment strategies in CLL.

## Introduction

B-cell chronic lymphocytic leukemia (CLL) is a heterogeneous disease characterized by clonal proliferation and the accumulation of mature CD5^+^ B lymphocytes in the bone marrow, peripheral blood, and lymphoid tissues (1). Although it has been suggested that memory B-cells give rise to CLL B-cells (2, 3), the latter’s cellular origin is still subject to debate. Nevertheless, identification of the normal counterpart is crucial for clarifying the pathogenesis, disease mechanism, and natural history of CLL. The observation that approximately half of all CLL patients carry somatically mutated immunoglobulin heavy-chain variable (IgHV) genes challenged the hypothesis in which CLL B-cells are derived from CD5^+^ B-cells (because the latter rarely have IgHV mutations) (2). It was then suggested that (i) mutated CLL B-cells were derived from B-cells that had undergone a germinal center reaction (i.e., post-germinal-center antigen-experienced B-cells) and (ii) unmutated CLL B-cells were derived from a pre-germinal center B-cells (2). Gene expression profiling shows that only a relatively small set of genes (20–100) can discriminate between mutated and unmutated CLL clones (3, 4). Mutated and unmutated CLL B-cells display a common, characteristic gene expression profile that is largely independent of their IgV genotype and is more strongly reminiscent of memory B-cells than of cells derived from naive B-cells, CD5^+^ B-cells, or germinal center centroblasts/centrocytes (3). Furthermore, the almost constant expression of CD27 seen in CLL cells has been linked to a memory phenotype (2, 5). Very recently, Seifert et al. performed transcriptome analyses of CLL and normal B-cell subsets. The researchers controversially reported that (i) unmutated CLL clones were derived from mature, unmutated CD5^+^CD27^−^ B-cells and (ii) mutated CLL clones were derived from a distinct, previously unrecognized, CD5^+^CD27^+^ post-germinal-center (memory) B-cell subset (6). To address the question of the CLL B-cells’ normal counterpart, most literature studies have used immunophenotypic, molecular, and gene expressing profiling to look for similarities between CLL B-cells and normal B-cells isolated *ex vivo*. Here, we propose a new way to address the question of the normal counterpart: the comparison of the phenotypic and functional features of antibody-secreting cells (ASCs) generated from CLL B-cells or from normal B-cells.

Terminal B-cell differentiation is a multistage process during which mature B-cells give rise to (i) short-lived ASC/plasma cells in an extrafollicular response and (ii) long-lived ASC/plasma cells and memory B cells after a germinal center reaction (7). Memory B-cells can survive for several months in the absence of antigenic stimulation and provide an early antibody response against recurrent infections (8, 9). In humans, between 30 and 40% of the B-cells in the peripheral blood are memory B-cells. There are two main types of memory B-cells: (i) immunoglobulin (Ig)-switched memory B-cells (CD27^+^IgD^−^IgG/A/E^+^) and (ii) non-switched IgM^+^ memory B-cells, which include IgM-only memory B-cells (CD27^+^IgM^+^IgD^−^) and a circulating subset of marginal zone B-cells CD27^+^IgM^+^IgD^+^ called IgM memory B cells, each of which accounts for about 15–20% of total B-cells (9–12). Recent studies have shown that IgM memory B-cells subset probably have a germinal center-independent origin; they undergo somatic hypermutation (SHM) during generation of the pre-immune repertoire and are solely involved in responses to T-independent antigens (11–13). It is noteworthy that patients with hyper-IgM syndrome type I [resulting from defects in the gene for CD40 ligand (CD40L)] have IgM memory B-cells (with SHMs) but not switched memory B cells (11, 12, 14). These patients are unable to generate germinal centers and have low or null serum IgG, IgA, and IgE levels and normal or elevated serum IgM levels (11, 12).

The Toll-like receptor (TLR) 9 is activated by CpG oligodeoxynucleotide (CpG) motifs present in unmethylated viral and bacterial DNA (15). In humans, the CpG ligand has a key role in the maintenance of memory B-cells and the establishment of long-term serological memory and natural antibody production (8, 15). *In vitro*, human memory B-cells differentiate into ASCs in response to polyclonal stimuli, bystander T cell help (CD40L) and CpG oligodeoxynucleotide (8, 15– 21). In fact, TLR9 (22, 23), CD40L–CD40 interactions (24, 25), and cytokines (26) are all important components of the CLL microenvironment. *In vitro*, CD40 stimulation of CLL B-cells results in NF-κB activation and is used as a model to mimic the lymph node microenvironment (25, 27). As with normal B-cells, CLL B-cells express a functional TLR9 (22, 23). Stimulation through the TLR9 activates CLL B-cells and induces an immunogenic phenotype (22, 23). It was recently suggested that studying TLR9 responses could provide further insight in the physiopathology of CLL and thus lead to the development of novel therapeutic strategies (28). Gutierrez et al. have shown that CpG induces CLL B-cells to differentiate into ASCs (29). However, these authors were not working with isolated CLL B-cells. Furthermore, they used a one-step culture system and did not characterize the immunophenotypic and molecular features of the generated ASCs. In the present study, we stimulated purified CLL B-cells with a combination of CpG, CD40L and cytokines (referred to hereafter as the “CpG/CD40L/c” condition) and investigated their ability to differentiate into ASCs/plasma cells in a 7-day, two-step culture model. We also characterized the cytomorphologic, immunophenotypic, molecular, and functional features of the resulting ASCs. Lastly, in order to functionally define a normal cellular counterpart for CLL B-cells, we tried to establish a link between the functional characteristic of ASCs generated from CLL B-cells and those generated from normal B-cell subpopulations (as described by literature studies that had used a similar culture system).

## Materials and Methods

### Patients

Chronic lymphocytic leukemia B-cells were obtained from the peripheral blood of 12 treatment-naive patients [three women and nine men; median (range) age: 67 (48–82)] diagnosed according to the international guidelines (Table 1). All patients displayed the clonal expansion of small, CD38-negative lymphocytes with high nucleus/cytoplasm ratios and co-expression of CD19, CD5, and CD23. In six of the 12 patients (# 3, 4, 6, 9, 10, and 12), mutational status of the IgHV genes was determined: three patients had unmutated IgHV genes (i.e., <2% mutations at the DNA level) and three patients had mutated IgHV genes (i.e., >2% mutations) (Table 1). Normal B-cells were isolated from the peripheral blood of healthy volunteers. All 12 patients and two healthy subjects provided their written informed consent to participation in the study. All procedures involving patient samples were approved by the local investigational review board (*Comité de Protection des Personnes Nord-Ouest*, Amiens, France).

**Table 1:** **Patient characteristics and VDJH rearrangements**.

Patient	Sex	Age	Binet stage	Matutes score	CD38	Cytogenetics	Mutational status	VH	DH – JH
1	M	65	A	5	–	13q14 del	ND	ND	ND
2	M	80	A	5	–	ND	ND	ND	ND
3	F	59	B	5	–	13q14 del	M (10.7%)	VH4–34	DH3–22 – JH4
4	M	65	A	5	–	Trisomy 12	UM	VH1–69	DH3–3 – JH3
5	F	80	A	5	–	ND	ND	ND	ND
6	M	82	A	5	–	NORMAL	M (8.3%)	VH3–33	DH4–17 – JH4
7	M	55	A	5	–	13q14 del	ND	ND	ND
8	M	76	A	5	–	Trisomy 12 Monosomy 9	ND	ND	ND
9	M	75	A	4	–	ND	UM	VH1–69	DH3–10 – JH5
10	M	48	B	5	–	13q14, 11q del	UM	VH3–49	DH3–3 – JH4
11	M	69	A	5	–	17p del	ND	ND	ND
12	F	68	A	4	–	ND	M (10.6%)	VH4–34	DH5–24 – JH4

*del: deletion; ND: not determined; M: mutated; UN: unmutated; VH DH and JH: heavy-chain variable, diversity, and joining region gene segments, respectively*.

### Cell isolation and culture

Peripheral blood mononuclear cells were isolated by Ficoll density gradient centrifugation of heparinized venous blood samples from CLL patients. CD19^+^CD5^+^ CLL B-cells were purified by negative selection using magnetic bead-activated cell sorting (MACS), with a B-cell (B-CLL) isolation kit (Miltenyi Biotec). The purity of all preparations was around 98% and the cells co-expressed CD19 and CD5 at their surface (as assessed by flow cytometry). Direct labeling with anti-CD2, CD14, and CD56 antibodies was always used to check that purified CLL B-cells were not contaminated by other immune cells. Cells were cultured in RPMI 1640 medium (Gibco-Invitrogen) [supplemented with 10% fetal calf serum (PAA), 100 IU/ml penicillin, 100 μg/ml streptomycin, and 2 mM l-glutamine] at 37°C in a 5% CO_2_ humidified incubator.

On day 0 (D0), purified CLL B-cells were seeded at a concentration of 2 × 10^6^/ml and stimulated for 4 days with phosphorothioate CpG oligodeoxynucleotide 2006 (10 μg/ml; Sigma-Aldrich) in association with histidine-tagged soluble recombinant human CD40L (50 ng/ml) and anti-polyhistidine monoclonal antibody (mAb) (5 μg/ml; R&D Systems) and a combination of interleukin (IL)-2 (50 ng/ml), IL-10 (50 ng/ml), and IL-15 (10 ng/ml). The cells were cultured in 5 ml in six-well, flat-bottomed culture plates.

On D4, the cells were harvested, washed, and seeded at a concentration of 10^6^/ml and cultured in the presence of IL-2 (50 ng/ml), IL-6 (50 ng/ml), IL-10 (50 ng/ml), and IL-15 (10 ng/ml) for 3 days. On D7, cells were harvested, washed, and analyzed. All human recombinant cytokines were purchased from PeproTech EC.

Normal B-cells were cultured as described previously (17).

### Immunophenotypic analysis

Cells were stained with appropriate combinations of fluorochrome-conjugated antibodies in direct immunofluorescence assays. The suppliers and specificities of the applied mAbs and the murine isotype-matched controls are given in Table S1 Supplementary Material. The Cytofix/Cytoperm kit (BD Biosciences) was used for intracellular staining of IgM, IgG, and CD38, according to the manufacturer’s instructions. Flow cytometry analysis was performed with a FACSCantoII flow cytometer (BD Biosciences). FlowJo software (Tree Star) was used for data analysis. Antigen density was expressed as the relative fluorescence intensity (RFI), i.e., the ratio between the mean fluorescence intensity (MFI) of cells labeled with a specific antibody and the MFI of cells labeled with a matched isotype control.

### Morphological analysis

For the morphological analysis on D7, cells were cytospinned (500 rpm, 5 min). Cytospin smears were stained with May-Grünwald–Giemsa (MGG) reagent and viewed under a light microscope (Axio Imager. M2; Zeiss) equipped with an AxioCam MRc5 microscope digital camera. Images were acquired using ZEN pro Software (Zeiss). We used PowerPoint software to render images with uniform backgrounds.

### Quantitative real-time PCR analysis

On D0 and D7, total RNA was isolated from cells using an RNeasy Mini kit (Qiagen). One microgram of total RNA was used for cDNA synthesis with a High Capacity cDNA Archive kit (Applied Biosystems). For transcript detection with quantitative real-time PCR (qRT-PCR), primers, probes, and TaqMan^®^ Universal PCR Master Mix were used according to the manufacturer’s instructions (Applied Biosystems) and PCRs were run on a StepOnePlus^TM^ Real-time PCR system (Applied Biosystems). The TaqMan^®^ Gene Expression Assays for *PRDM1* (BLIMP1) (Assay ID Hs00153357_m1), *PAX5* (Hs00172003_m1), *EBF1* (Hs00395524_m1), *BCL6* (Hs00277037_m1), *XBP1s* (Hs 03929085_g1), *IRF4* (Hs01056533_m1), *IRF8* (Hs01128710_m1), *BACH2* (Hs00222364_m1), *GAS6* (Hs01090305_m1), *AICDA* (Hs00757808_m1), *IGHA1* (Hs00733892_m1), *IGHG1* (Hs00378 340_m1), *CD38* (Hs01120071_m1), and *CD138* (Hs00896423_ m1) were purchased from Applied Biosystems. For quantitation, β2-microglobulin (B2M, 4333766F) was used as an endogenous control. Variations in mRNA expression were calculated using the 2^−ΔΔCT^ qRT-PCR method, where ΔΔCT = ΔCT D7 − ΔCT D0.

For growth-arrest-specific gene 6 (*GAS6*), *AICDA*, *IGHG1*, and *IGHA1* the change in mRNA expression was determined using the 2^–ΔCT^ method, where ΔCT = CT of target gene – CT of B2M.

### Analysis of IgM, IgG, and IgA secretion

The levels of human IgM, IgG, and IgA in the culture supernatants were quantified with the appropriate ELISA kit (Bethyl Laboratories). Immunoglobulin production (in micrograms per 10^6^ cells) was estimated by dividing the total amount of Ig in the culture supernatant by the number of live cells.

### Indirect immunofluorescence assays

Slides coated with HEp-2 cells (INOVA Diagnostics) were incubated with culture supernatant for 1 h at room temperature, washed in PBS, incubated with an FITC-conjugated anti-human IgM antibody and viewed under a fluorescence microscope (Axio Imager M2; Zeiss) equipped with an AxioCam MRc5 microscope digital camera. Images were acquired with ZEN pro software (Zeiss). Positive controls (serum samples from patients with the autoimmune disease scleroderma) and negative controls (culture medium) were included in all experiments. The term poly/autoreactivity was used to indicate (i) autoreactivity (when staining was positive) and (ii) polyreactivity (when several cell components stained positive – the nucleus and cytoplasm, for example).

### Clonality assessment, V(D)J sequencing, and somatic hypermutations analysis

For CLL samples (# 3, 4, 6, 9, 10, and 12), genomic DNA was extracted using the QIAamp spin column technology (Qiagen). Immunoglobulin heavy-chain (IgH) and immunoglobulin light chain (IgL) gene rearrangements were analyzed in a multiplex PCR using the standardized BIOMED-2 PCR protocol (30). The PCR products were electrophoretically separated on a 3500xL Dx Genetic Analyzer (Applied Biosystems) and size analysis was performed using GeneMapper^®^ Software v4.1. For the size analysis, 1 μl of PCR product was mixed with 0.5 μl of a dye-labeled size standard (GeneScan™ 500 LIZ™ dye Size Standard, Applied Biosystems) and 12 μl of deionized formamide (Hi-Di™ Formamide, Life Technologies). The mixture was heated at 95°C for 1 min prior to microcapillary electrophoresis. Monoclonality was defined as one or two peaks of amplified PCR products in a GeneScan analysis. For the analysis of V (D), and J sequences, approximately 50 ng of the purified PCR product were sequenced using a BigDye^®^ Terminator v1.1 Cycle Sequencing Kit (Applied Biosystems), according to the manufacturer’s instructions. Electropherograms were analyzed with Sequencing Analysis v.5.4 software (Applied Biosystems) and sequence data were analyzed using the international ImMunoGeneTics information system^®^ (IMGT^®^, http://www.imgt.org) (31) and the Basic Local Alignment Search Tool (BLAST) database. The mutation rate in the rearranged IgVH gene was defined as the percentage of mutations per VH sequence, after sequencing and detection of mutations in both the sense and antisense strands (Table 1).

### Statistical analysis

All statistical analyses were performed with Prism 5 software (GraphPad Software). The statistical significance of intergroup differences was determined using the Wilcoxon test or Student’s *t*-test, as appropriate; *p* values below 0.05 were considered to be statistically significant and *p* values below 0.01 were considered to be highly statistically significant. Significant differences are denoted as follows: *p < 0.05, **p < 0.01, and ***p < 0.001.

## Results

### CLL B-cells and normal B-cells respond differently in the CpG/CD40L/c culture system

**Figure 1:**
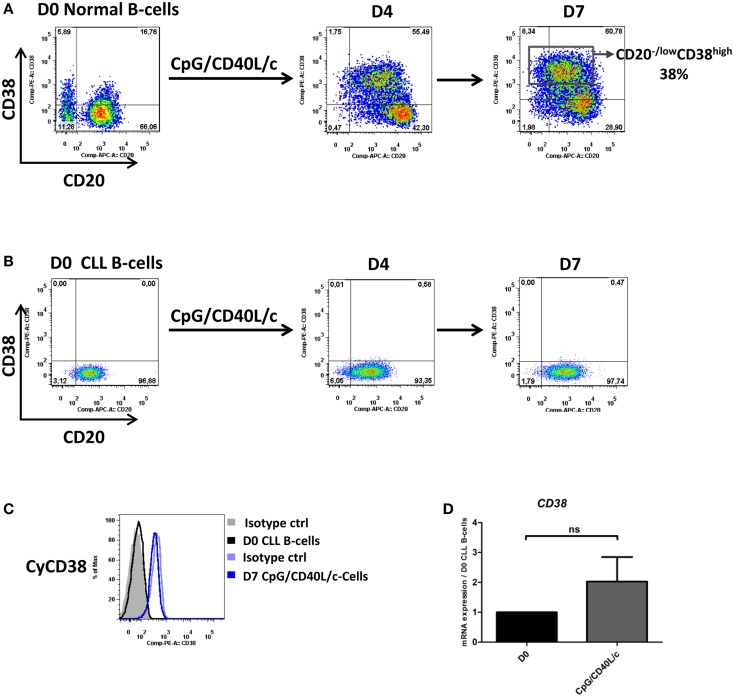
**CD20 and CD38 expression on normal and CLL B-cells**. Cells were stimulated with CpG, CD40L, and the cytokines IL-2, IL-10, and IL-15. On day 4 (D4), cells were harvested and incubated with IL-2, IL-6, IL-10, and IL-15 for 3 days. **(A,B)** Cells were labeled with anti-CD20 and anti-CD38 mAbs to examine changes in surface expression on normal B-cells **(A)** and CLL B-cells **(B)** on D0, D4, and D7. Cytometry plots are representative of two and nine independent experiments for normal B-cells and CLL B-cells, respectively. **(C)** Cells were labeled with anti-CD38 after permeabilization. Cytometry plots of cytoplasmic CD38 are representative of four experiments. **(D)** Expression of the CD38 gene was evaluated using qRT-PCR in D0 CLL B-cells and D7-stimulated cells. The results are expressed relative to the gene expression in CLL B-cells on D0, according to the 2^−ΔΔCT^ method. Bars represent mean ± SEM values from five experiments. Statistical significance was calculated in a Wilcoxon test. ns, not significant. D, Day. Cy, Cytoplasmic.

To validate the effectiveness of the CpG culture system, we used normal B-cells from healthy donors as a positive control [as described in Ref. (17)]. As shown in Figure 1A, the expression of CD38 (a marker of B-cell activation and plasma cell differentiation) was increasingly expressed on the surface of normal B-cells at each step in the CpG/CD40L/c culture system (it increases from 18 ± 4.2% at D0 to 70 ± 8% at D7). On D7, the appearance of a population of CD20^low/−^CD38^high^ cells (it increases from 1.2 ± 0.5% at D0 to 40 ± 2.8% at D7) confirmed that the CpG/CD40L/c culture system was working properly (Figure 1A).

When compared with normal B-cells, CLL B-cells differed in their response under the same culture conditions (Figure 1B). On D0, the cells were CD20^+^CD38^−^. On D7, we first noted the absence of marked surface CD38 expression in CpG/CD40L/c-stimulated cells (Figures 1A,B). In order to establish whether CD38 was expressed but not directed to the cell surface, we checked for cytoplasmic CD38 and CD38 mRNA expression. As shown in Figures 1C,D, CD38 expression was absent in the cytoplasm and mRNA levels were only slightly upregulated on D7.

**Figure 2:**
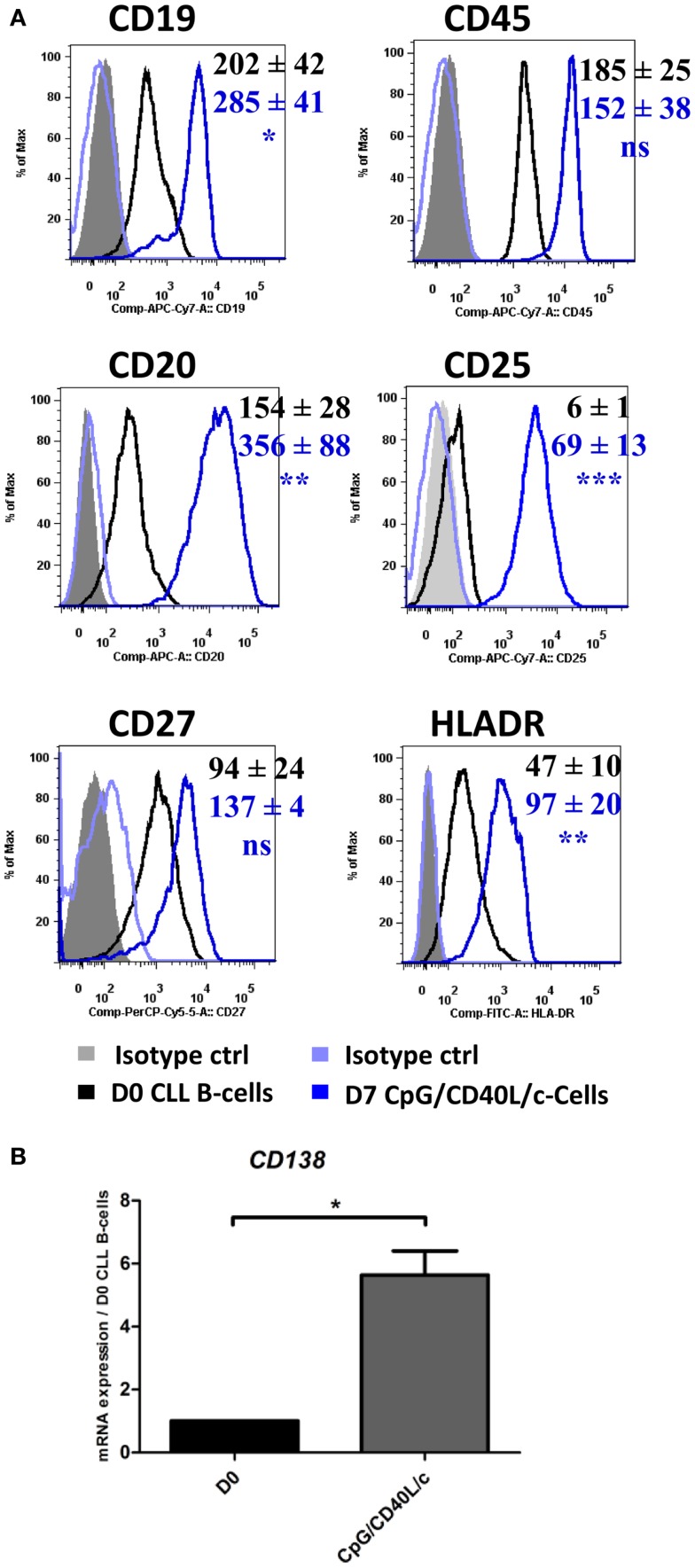
**The immunophenotype of the generated cells on D7**. On D0 and D7, the cell immunophenotype was studied by direct labeling of CD19, CD20, CD27, CD45, CD25, and HLADR. **(A)** Relative fluorescence intensities (RFIs) were calculated as the ratio between the mean fluorescence intensity (MFI) of cells labeled with a specific antibody and the MFI of cells labeled with a matched isotype control. Mean ± SEM RFI values from nine experiments are represented with a color code: black: D0 values, blue: D7 CpG/CD40L/-stimulated cells. Cytometry data are presented as plots from a representative patient. **(B)** The expression of the CD138 gene was evaluated with qRT-PCR in D0 CLL B-cells and D7-stimulated cells. Results are expressed relative to gene expression in CLL B-cells on D0, according to the 2^−ΔΔCT^ method. Bars represent the mean ± SEM values from five experiments. Statistical significance was calculated in a Wilcoxon test. *The D7 value differs from the D0 value in CLL B-cells. *p < 0.05, **p < 0.01, and ***p < 0.001. ns, not significant. D, Day.

In order to investigate possible changes in the immunophenotype on D7, cells were stained for CD19, CD20, CD27, CD45, CD25, CD138, and HLADR (Figure 2A). CpG/CD40L/c-stimulated cells significantly upregulated their expression of CD20 and CD19 on D7, whereas CD27 and CD45 expression did not change significantly. On D7, the cells also significantly upregulated their expression of HLADR and CD25 (Figure 2A). Although cell surface CD138 expression was not detected (data not shown), CD138 mRNA levels were significantly upregulated (Figure 2B).

### Day-7-generated cells develop an ASC/plasma cell morphology

**Figure 3:**
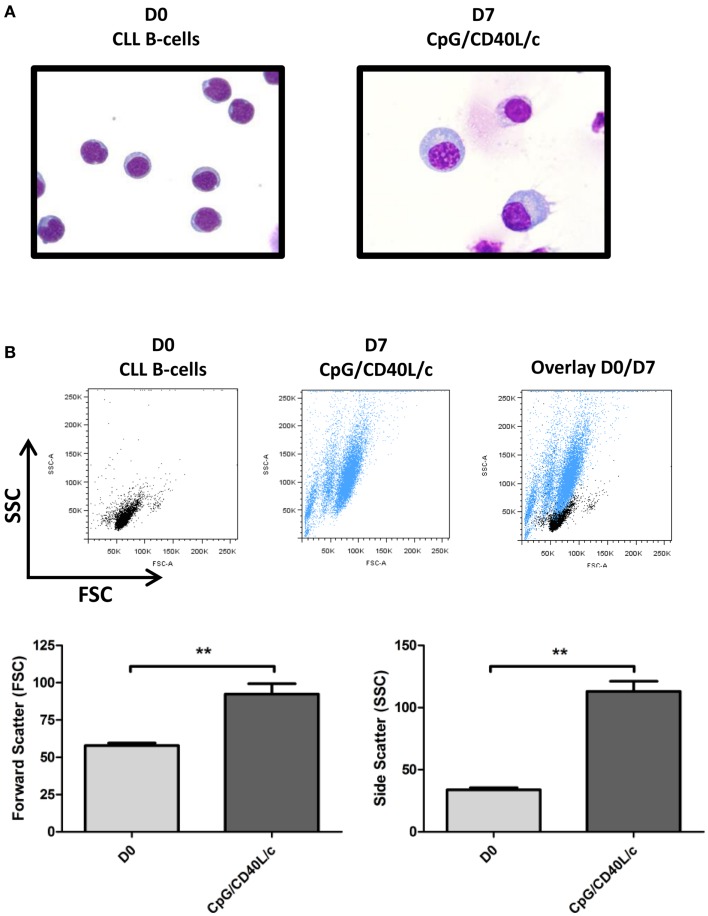
**Morphological analysis of D0 CLL B-cells and D7-stimulated cells, as revealed by MGG staining and flow cytometry**. **(A)** CLL B-cells (D0) and stimulated cells (D7) were stained with MGG reagent. Original magnification: ×1000. **(B)** Cell size and granularity were measured by flow cytometry. The relative cell size is determined by light diffracted at small angles [detected as forward scatter (FSC)]. The granularity is proportional to the light diffracted at large angles [detected as side scatter (SSC)]. The statistical significance was calculated in a Wilcoxon test: **p < 0.01.

As shown by MGG staining (Figure 3A), D0 CLL B-cells presented a normal-shaped nucleus that was surrounded by a thin ring of cytoplasm. On D7, CpG/CD40L/c-stimulated cells showed a typical ASC/plasma cell morphology, i.e., an eccentric nucleus and abundant cytoplasm. Consistent changes in cell morphology were also monitored by flow cytometry, on the basis of the cells’ relative size (forward scatter, FSC) and granulometry (side scatter, SSC) (Figure 3B).

### Day-7-generated cells express simultaneously plasma cell and B-cell transcription factors

**Figure 4:**
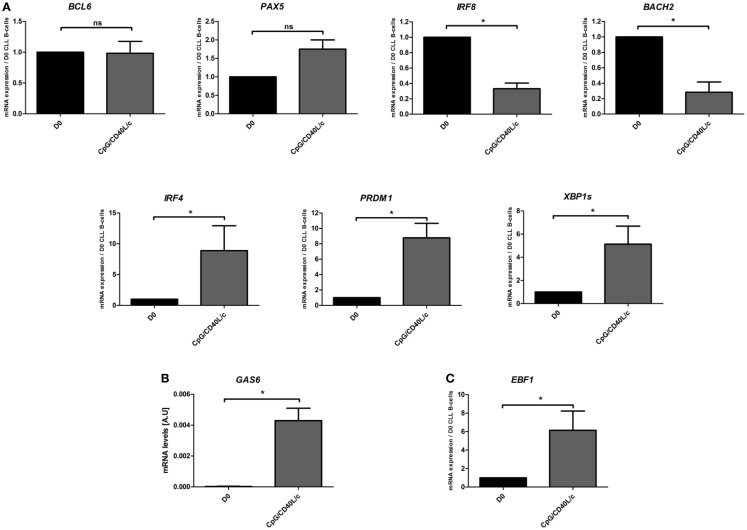
**Day 7 mRNA expression analysis of transcription factors involved in B-cell-to-plasma-cell differentiation**. **(A,C)** The transcriptional expression of *BCL6*, *PAX5*, *BACH2*, *IRF8*, *IRF4*, *PRDM1*, *XBP1s*, and *EBF1* genes was evaluated in a qRT-PCR on D0 and D7. The results are expressed relative to gene expression in CLL B-cells on D0, according to the 2^−ΔΔCT^ method. Data are expressed as the mean ± SEM from five experiments. **(B)** The relative mRNA expression of *GAS6* in CLL B-cells on D0, compared with CpG/CD40L/c-stimulated cells on D7. The data were calculated according to the relative 2^–ΔCT^ method. The values on D7 were compared with those on D0, and the statistical significance was calculated in a Wilcoxon test: *p < 0.05. ns, not significant.

To further understand phenotypic changes in D7-generated cells, we studied the mRNA expression of several transcription factors that are found in B-cells [PAX5, BCL6, BACH2, IRF8, and early B-cell factor 1 (EBF1)] and are known to be involved in plasma cell differentiation [IRF4, PRDM1, and X-box binding protein 1 spliced form (XBP1s)] (Figure 4). On D7, we observed that CpG/CD40L/c-stimulated cells displayed a significant increase in transcription of the *IRF4*, *PRDM1*, *XBP1s* genes and a significant decrease in the transcription of the *BACH2* and *IRF8* genes (Figure 4A). However, mRNA expression of *PAX5* and *BCL6* was not affected (Figure 4A). Moreover, mRNA expression of growth-arrest-specific gene 6 (*GAS6*) (Figure 4B) and *EBF1* was significantly induced on D7 (Figure 4C).

### Mutated and unmutated CLL B-cells differentiate into IgM-secreting cells

**Figure 5:**
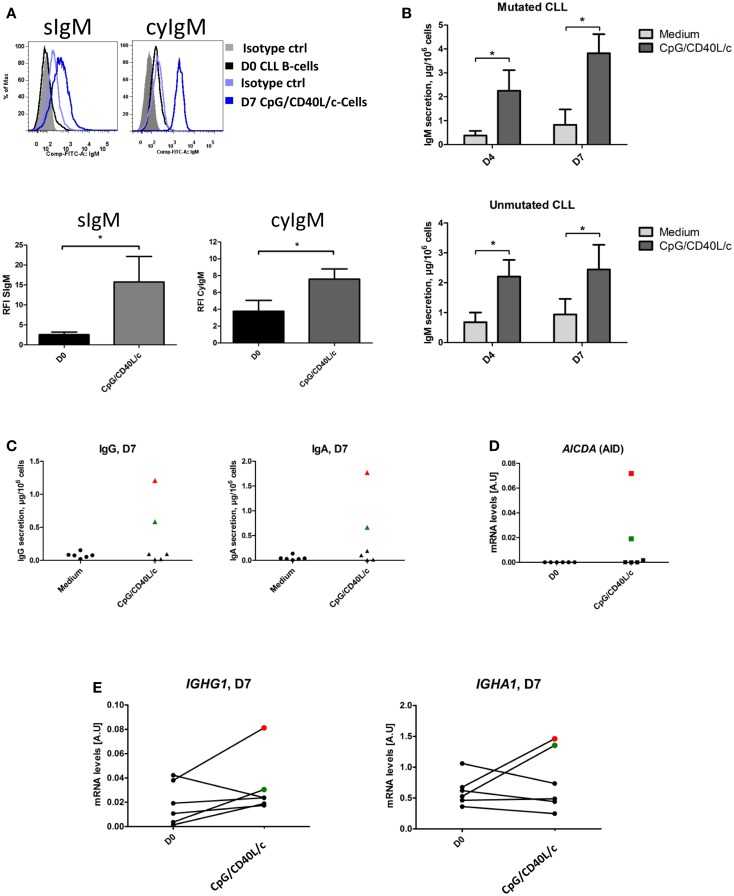
**Ig expression and secretion by CpG/CD40L/c-stimulated cells**. **(A)** CLL B-cells (on D0) and CpG/CD40L/c-stimulated cells (on D7) were labeled before and after permeabilization with FITC-conjugated anti-human IgM mAbs or isotype-control mAbs. Cytometry plots are representative of four experiments. S, Surface, Cy, cytoplasmic. The bar histograms show the relative fluorescence intensity (RFI) of surface and cytoplasmic IgM. The RFI was calculated as the ratio between the mean fluorescence intensity (MFI) of cells labeled with a specific antibody and the MFI of cells labeled with a matched isotype control. Data are quoted as the mean ± SEM of four experiments. **(B)** Culture supernatants were harvested on D4 and D7. IgM secretion was assessed with an ELISA. The results are quoted as the mean ± SEM (in micrograms per 10^6^ cells) from three mutated CLL samples and three unmutated CLL samples. **(C)** Culture supernatants were harvested on D7. Secreted IgG and IgA levels were assessed with an ELISA. The red and green point correspond to CLL samples #4 and #10, respectively. **(D)** The relative expression of *AICDA* in CLL B-cells on D0, compared with CpG/CD40L/c-stimulated cells on D7. The data were calculated according to the relative 2^–ΔCT^ method. **(E)** The transcriptional expression of IGHG1 and IGHA1 in CLL B-cells on D0, compared with CpG/CD40L/c-stimulated cells on D7. Quantitative real-time PCR analysis was performed on total RNA extracted from cells at D0 and D7. IGHG1 and IGHA1 mRNA expression are upregulated in cells in which IgG and IgA class-switch recombination occurred [the red (CLL#4) and green (CLL#10) points].The statistical significance was calculated in a paired *t*-test: *p < 0.05.

**Figure 6:**
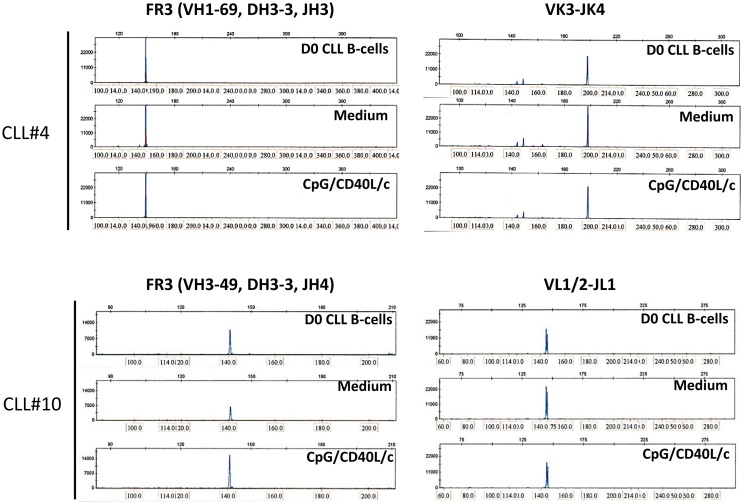
**GeneScan analysis of IgH and IgL gene rearrangements shows a monoclonal pattern at D0 and D7**. Fragments are aligned by size (indicated above and at the bottom of each panel). Left panels show a peak in the IgH framework 3 (FR3)-specific size range for D0 CLL B-cells (upper panel), D7 untreated-cells (Medium, middle panel), and D7 CpG/CD40L/ c-stimulated cells (lower panel), indicating a monoclonal rearrangement. Right panels show a peak in the IgL-specific size range for D0 CLL B-cells, D7 untreated-cells (medium), and D7 CpG/CD40L/c-stimulated cells, indicating a monoclonal rearrangement. The data presented are for CLL samples #4 and #10, in which isotype-switching was detected. The data are representative of six experiments (CLL samples #3, #4, #6, #9, #10, and #12)

To establish whether the stimulation conditions induced CLL B-cells to differentiate into ASCs, we assessed the cytoplasmic expression and secretion of Igs in three mutated CLL samples and three unmutated CLL samples. On D0, IgM was absent from the surface, or present in only small amounts (Figure 5A). On D7, IgM expression on the cell surface was significantly upregulated (Figure 5A). On D0, all the CLL B-cells expressed cytoplasmic IgM. On D7, cytoplasmic expression of IgM was upregulated (Figure 5A). We then used ELISAs to investigate IgM, IgG, and IgA secretion into the culture supernatant on D4 and D7 (Figure 5B). When compared with cell culture in the absence of stimulation (i.e., in medium only), CpG/CD40L/c-stimulated mutated and unmutated CLL B-cells secreted significant amounts of IgM. Considerable amounts of secreted IgM could be detected as early as D4 (Figure 5B). Moreover, the presence of at least moderate levels of IgG and IgA revealed that class-switch recombination (CSR) was triggered in two of the three unmutated CLL samples (and none of the mutated samples) (Figure 5C). Expression of *AICDA* mRNA was detected in cells in which CSR was observed (Figure 5D). Furthermore, gamma and alpha H-chain transcripts were upregulated in the two CLL samples with CSR (Figure 5E). To check whether or not the IgA and IgG were being secreted by contaminating, residual, normal B cells, we used PCR DNA sequencing and high-resolution PCR fragment analysis (GeneScan) to study Ig light and heavy-chain gene rearrangements and monoclonality. The fragment analysis showed that cells were always clonal after differentiation on D7 (Figure 6). Sequencing of the CDR3 regions showed that the sequences were identical at D0 and D7 (data not shown).

### IgMs secreted by ASCs derived from unmutated CLL B-cells are poly/autoreactive

**Figure 7:**
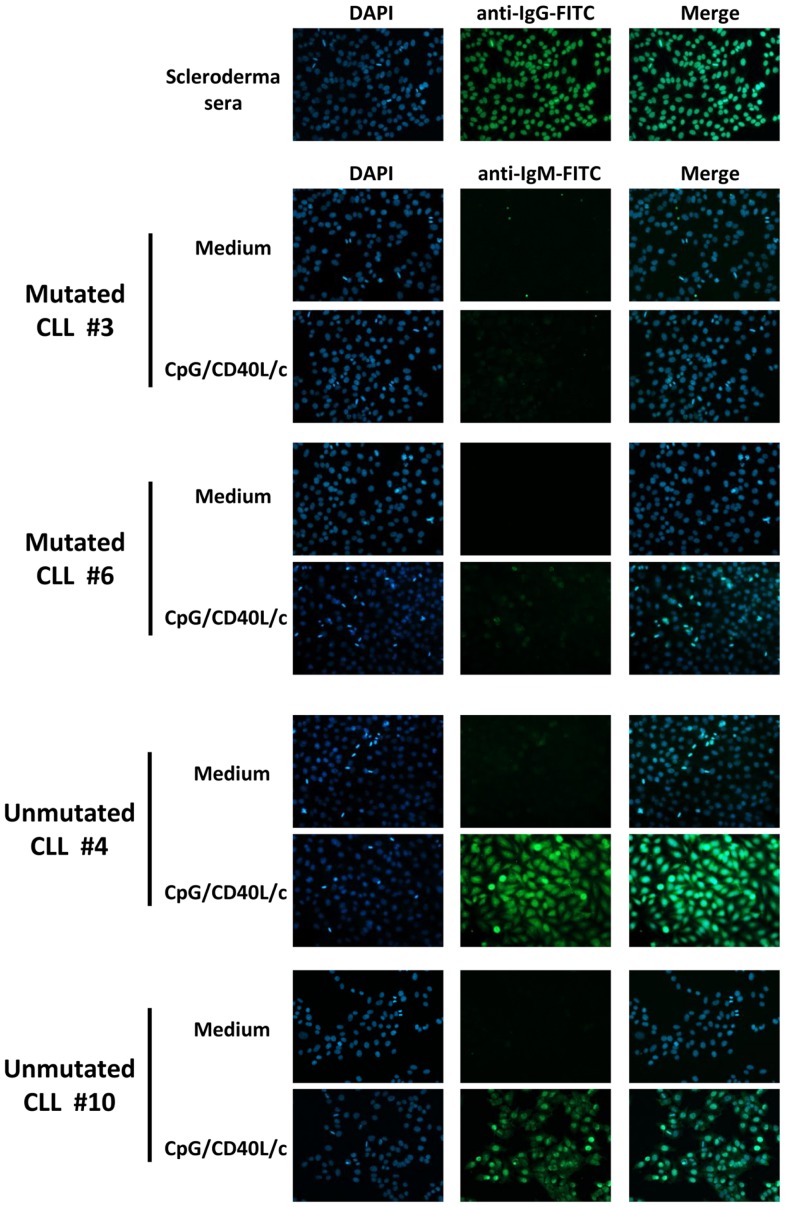
**Poly/autoreactivity of secreted IgM, as evaluated by immunofluorescence staining of HEp-2 cells**. Day 7 supernatants from six experiments (three mutated CLL samples and three unmutated CLL samples) were tested for reactivity with HEp-2 cells. Serum from scleroderma was used as a positive control for autoreactivity. Indirect immunofluorescence analysis showed that the IgM secreted into the culture supernatant by ASCs having differentiated from unmutated CLL B-cells were able to bind to autoantigens in HEp-2 cells. Magnification: ×200.

To determine whether the secreted IgM antibodies were poly/autoreactive, we performed indirect immunofluorescence assays on slides coated with HEp-2 cells (Figure 7). We found that the IgMs secreted by ASCs derived from unmutated CLL B-cells displayed a cytoplasmic or nuclear/cytoplasmic staining pattern (Figure 7). In contrast, IgMs secreted by ASCs derived from mutated CLL B-cells were not poly/autoreactive (Figure 7).

## Discussion

In the present study, we looked at whether CpG stimulation in the context of CD40 ligation was able to induce CLL B-cells to differentiate into ASCs/plasma cells in a 7-day, two-step culture system. Differentiation into ASCs/plasma cells involves profound phenotypic, molecular and morphologic changes, as mature B-cells are transformed into cells capable of producing large amounts of antibodies (7). Classically, B-cell differentiation leads to the downregulation of B-cell surface markers (such as CD19 and CD20), the expression of plasma cell markers (such as CD38 and CD138), and a switch from surface-displayed Igs to cytoplasmic/secreted Igs. These phenotypic changes correlate with decreased expression of B-cell transcription factors (such as PAX5, BCL6, IRF8, and BACH2), the expression of plasma cell transcription factors (such as IRF4, BLIMP1, and XBP1) and the acquisition of a plasma cell morphology (e.g., an eccentric nucleus and the abundant cytoplasm required for antibody secretion) (7).

Our data show that when stimulated with CpG/CD40L/c, CLL B-cells differentiated into CD20^+^CD27^+^CD38^−^ASCs, which were able to produce large amounts of IgM. Importantly, we evidenced significant upregulation of mRNA levels for the plasma cell marker CD138. However, human ASCs/plasma cells have a heterogeneous phenotype (32, 33) that is related to their exact anatomic site and their degree of maturation; indeed, human tonsillar plasma cells were shown to express CD20 but not the plasma cell marker CD138 (32, 34, 35). Moreover, CD20^+^ human tonsillar plasma cells were shown to express both BLIMP1 and PAX5 (32). However, it was suggested that the differential expression of human CD38, CD138, HLADR, and CD20 may correspond to alternative or additional ASC subsets derived from independent differentiation pathways in blood, bone marrow, and possibly other tissues (33). Hence, the molecular and immunophenotypic signature of these ASCs may reflect a transitional stage during plasma cell differentiation. Nevertheless, the cells’ phenotype can be explained by the modulation of transcription factor expression. We noted the induction of mRNA expression of the early B-cell factor 1 (EBF1) transcription factor, which is known to regulate the expression of the *PAX5* gene (36). In our experiments, *PAX5* expression was slightly upregulated in CpG/CD40L/c-stimulated cells. Indeed, PAX5 and EBF1 have been shown to induce and regulate the expression of CD19 and CD20 in B-cells (36, 37). Moreover, expression levels of the *BCL6* gene (encoding a transcription factor required for germinal center formation and the maintenance of a centroblast phenotype (38)) did not change during the cell culture. Data from our qRT-PCR experiments also revealed the upregulation of plasma cell transcription factors *PRDM1* (BLIMP1), *IRF4*, and *XBP1s* (7, 38) and downregulation of B-cell transcription factors *BACH2* and *IRF8* (39). BACH2 is a PRDM1 inhibitor and so BACH2 downregulation enhances BLIMP1 expression (39). Furthermore, it is known that IRF4 induces BLIMP1 and XBP1 expression, indeed, BLIMP1 is required for plasma cell formation and the subsequent production of high levels of Ig (7, 40). X-box binding protein 1 (XBP1) is a transcription factor involved in plasma cell differentiation. It has a role in the unfolded protein response (UPR) that is essential for the secretion of large amounts of Ig by plasma cells (41). Only the spliced form of XBP1 (XBP1s) can activate the UPR efficiently (42). In our experiments, cells showed significant induction of GAS6 mRNA on D7. GAS6 is a member of the vitamin K-dependent protein family. Gene expression profiling data has shown that GAS6 is more strongly expressed in plasmablasts and plasma cells than in mature B-cells (43). GAS6 mRNA is present at negligible levels in precursor B-cells and B-cell lines but is detected in terminally differentiated plasma cell lines (44). Taken as a whole, our data show that CpG/CD40L/c activation induces CLL B-cells to differentiate into CD20^+^ IgM-secreting cells with an alternative immunophenotype and molecular profile.

CpG/CD40L/c stimulation clearly triggered CSR because at least moderate levels of IgG and IgA were detected in two of the six CLL samples. Interestingly, CSR was observed in two of the three unmutated CLL B-cell samples but not in any of the three mutated CLL B-cell samples. The presence of CSR was also confirmed by the upregulated expression of *AICDA* mRNA and gamma and alpha H-chain transcripts. Indeed, CLL B-cells were shown to undergo CSR *in vivo* (45). Similar results have been obtained *in vitro* by other researchers (46). Importantly, unmutated CLL B-cells were shown to express activation-induced cytidine deaminase and undergo CSR but not SHM (47, 48). SHM occurs in the variable region of the Ig heavy-chain and allows production of high-affinity, less autoreactive antibodies. Importantly, a great number of unmutated CLL B-cells express poly/autoreactive antibodies (49–51). In agreement with a previous study (49), our data show that only IgMs secreted by ASCs derived from unmutated CLL B-cells are poly/autoreactive.

Several studies have assessed the features of various B-cell subpopulations after different types of *in vitro* stimulation. The CD40 system induces a small proportion of naive B-cells to differentiate into IgM-secreting cells (few of which go on to produce IgG and IgA) (20, 21, 52). Under the same stimulation conditions, many CD27^+^ memory B-cells differentiate into predominantly IgG-secreting ASCs (8, 18, 20, 21, 52). Bernasconi et al. (8) reported that switched and unswitched memory B-cells respond differently to CpG and to CD40L. The researchers showed that in contrast to switched CD27^+^ memory B-cells, IgM^+^CD27^+^ memory B-cells respond efficiently to CpG oligodeoxynucleotide but not to CD40L (8). Indeed, the IgM^+^IgD^+^CD27^+^ subpopulation isolated from patients with hyper-IgM syndrome type I was found to secrete moderate levels of IgM in responses to CD40L stimulation in the presence of IL-4 or IL-10 (14, 53). However, our previous work has shown similar results, with low IgM levels when CLL B-cells are cultured in CD40 system (54). When triggered by CpG *in vitro*, memory B-cell differentiation usually gives rise to Ig-switched CD20^−^ASCs (16, 17). However, Geffroy-Luseau et al. (18) reported the generation of CD20^+^ ASCs from memory B-cells and suggested that CpG preferentially stimulates a subpopulation of IgM^+^ memory B-cells. Our data are similar to those of Geffroy-Luseau et al (18) and show that CpG activation induces CLL B-cells to differentiate into IgM-producing CD20^+^ ASCs. Moreover, IgM^+^IgD^+^CD27^+^ B-cells differentiate into ASCs in a CpG-based stimulation system and produce IgM and small amounts of IgG (19); in contrast, naïve B-cells do not generate plasma cells (19). Human blood naive CD27^−^B-cells do not express TLRs and require stimulation through their B-cell receptor (BCR) to express TLR9 and become responsive to CpG stimulation (55). When combined with BCR ligation and CD40L stimulation, CpG induces the differentiation of naïve B cells into ASCs that produce predominantly IgG but also some IgM and IgA (55). Activation with CpG (in the presence or absence of CD40L) triggers peripheral blood CD27^+^ memory B cells to produce primarily IgG but also IgM and IgA (16, 17). CLL B-cells were shown to differentiate into predominantly IgM-producing ASCs when stimulated with polyclonal B-cell activators (56, 57). Hence, CLL B-cells can differentiate into ASCs that secreted IgM when stimulated with CpG alone (29) and in the CpG/CD40L culture system (as shown in the present study). These data indicate that CLL B-cells originate from a B-cell compartment that differentiates into predominantly IgM-producing ASCs. However, in our experiments, only unmutated CLL B-cells underwent CSR and produced IgA and IgG. The IgMs produced by these cells were poly/autoreactive – indicating that the B-cell compartment can undergo CSR occasionally and generate poly/autoreactive antibodies. However, both SHM and CSR are by no means restricted to germinal center sites, and can occur ectopically. Human IgM memory B-cells do not originate from the germinal center (9, 11, 12); they carry immunoglobulins with low-frequency SHM, produce IgM (but also some IgG and IgA, after *in vitro* differentiation) and are considered to be the main source of “natural” antibodies in the body (9, 12). Moreover, human IgM memory B-cells share several functions and phenotypic characteristics with mouse B-1a and marginal zone B-cells (9, 12). In view of these findings, we hypothesize that both unmutated and mutated CLL B-cells are derived from IgM memory B-cells. IgM memory B-cells initially exhibit a “natural,” quasi-germinal IgHV repertoire, and then increase their specificity by undergoing SHM upon antigen encounter and recruitment in the immune response (9, 12, 13). Thus, the differences in mutational status between CLL cells would be unsurprising if (i) unmutated CLL B-cells are derived from less “antigen-experienced” IgM memory B-cells with little or no SHM [accounting for around 10% of IgM memory B-cells (9, 13)], and (ii) mutated CLL B-cells are derived from well “antigen-experienced” IgM memory B-cells with a high SHM frequency. It is noteworthy that human IgM memory B-cells have a heterogeneous phenotype and include both a CD5^+^CD27^+^ subpopulation (6) and a mutated CD27^−^ subpopulation (58). Taken as whole, our observations suggest that IgM memory B-cells might well be the normal counterpart of CLL B-cells. However, validation of this hypothesis will require further research.

Lastly, our data may help to improve treatment strategies for CLL. As we and others have shown, CpG treatment of CLL B-cells induces the upregulation of CD20 expression (59). CD20 is the target of rituximab and other antibody-based therapies under development (60). It is assumed that CD20 expression is low in CLL cells. This weak CD20 expression may be responsible for the lack of response to anti-CD20 therapy observed in certain patients. Increasing CD20 expression on the surface of CLL cells might increase the sensitivity of these cells to the pro-apoptotic effects of anti-CD20 antibodies. Furthermore, it has been suggested that differentiation therapy could be a promising way of treating CLL (29). Moreover, rituximab has been shown to specifically deplete autoreactive CD20^+^ plasma cells in a mouse model of inflammatory arthritis (35). We speculate that the use of CpG/CD40L to induce the differentiation of CLL B-cells might improve outcomes in the treatment of CLL.

## Author Contributions

Hussein Ghamlouch, Brigitte Gubler, and Jean-Pierre Marolleau designed the study; Hussein Ghamlouch, Hakim Ouled-Haddou, Aude Guyart, Aline Regnier, Vincent Fuentes, Jean-François Claisse, Stéphanie Trudel performed experiments and analyzed results; Jean-Pierre Marolleau, Brigitte Gubler, Stéphanie Trudel, and Bruno Royer supervised the study; Hussein Ghamlouch, Brigitte Gubler, and Jean-Pierre Marolleau wrote the manuscript.

## Conflict of Interest Statement

The authors declare that the research was conducted in the absence of any commercial or financial relationships that could be construed as a potential conflict of interest.

## Supplementary Materials

The Supplementary Material for this article can be found online at http://www.frontiersin.org/Journal/10.3389/fimmu.2014.00292/abstract
