# Supplementary material for: TLR9 Ligand (CpG Oligodeoxynucleotide) Induces CLL B-Cells to Differentiate into CD20+ Antibody-Secreting Cells
Source: Front Immunol. 2014 Jun 16;5:292. doi: 10.3389/fimmu.2014.00292 (PMC4058906; doi:10.3389/fimmu.2014.00292)
Supplement: Supplementary file 1 [file DataSheet_1.DOCX]

| Antigen | Clone | Source |
| --- | --- | --- |
| CD19-APC-Cy7 | SJ25C1 | BD Biosciences |
| CD20-APC | L27 | BD Biosciences |
| CD5-PerCP-Cy5.5 | L17F12 | BD Biosciences |
| CD38-PE | HIT2 | BD Biosciences |
| CD27-PerCP-Cy5.5 | M-T271 | BD Biosciences |
| CD45-APC-Cy7 | 2D1 | BD Biosciences |
| CD138-FITC | MI15 | BD Biosciences |
| HLADR-FITC | B8.12.2 | Beckman Coulter |
| CD25-APC-Cy7 | M-A251 | BD Biosciences |
| CD14-PE | M5E2 | BD Biosciences |
| CD2-FITC | 39C1.5 | Beckman Coulter |
| CD56-APC | B159 | BD Biosciences |
| IgM-FITC | G20-127 | BD Biosciences |
| IgG-FITC | G18-145 | BD Biosciences |

**Supplementary Table 1. List of antibodies used in immunophenotyping**
